# Supplementary material for: Catabolism of extracellular glutathione supplies cysteine to support tumours
Source: Nature. 2026 Mar 18;653(8115):933–41. doi: 10.1038/s41586-026-10268-2 (PMC13190318; doi:10.1038/s41586-026-10268-2)
Supplement: Supplementary file 1 — Uncropped western blot membranes corresponding to Figs. 1f and 3a. [file 41586_2026_10268_MOESM1_ESM.pdf]

nature portfolio

https://doi.org/10.1038/s41586-026-10268-2

Supplementary information

Catabolism of extracellular glutathione supplies cysteine to support tumours

In the format provided by the authors and unedited

Supplementary information

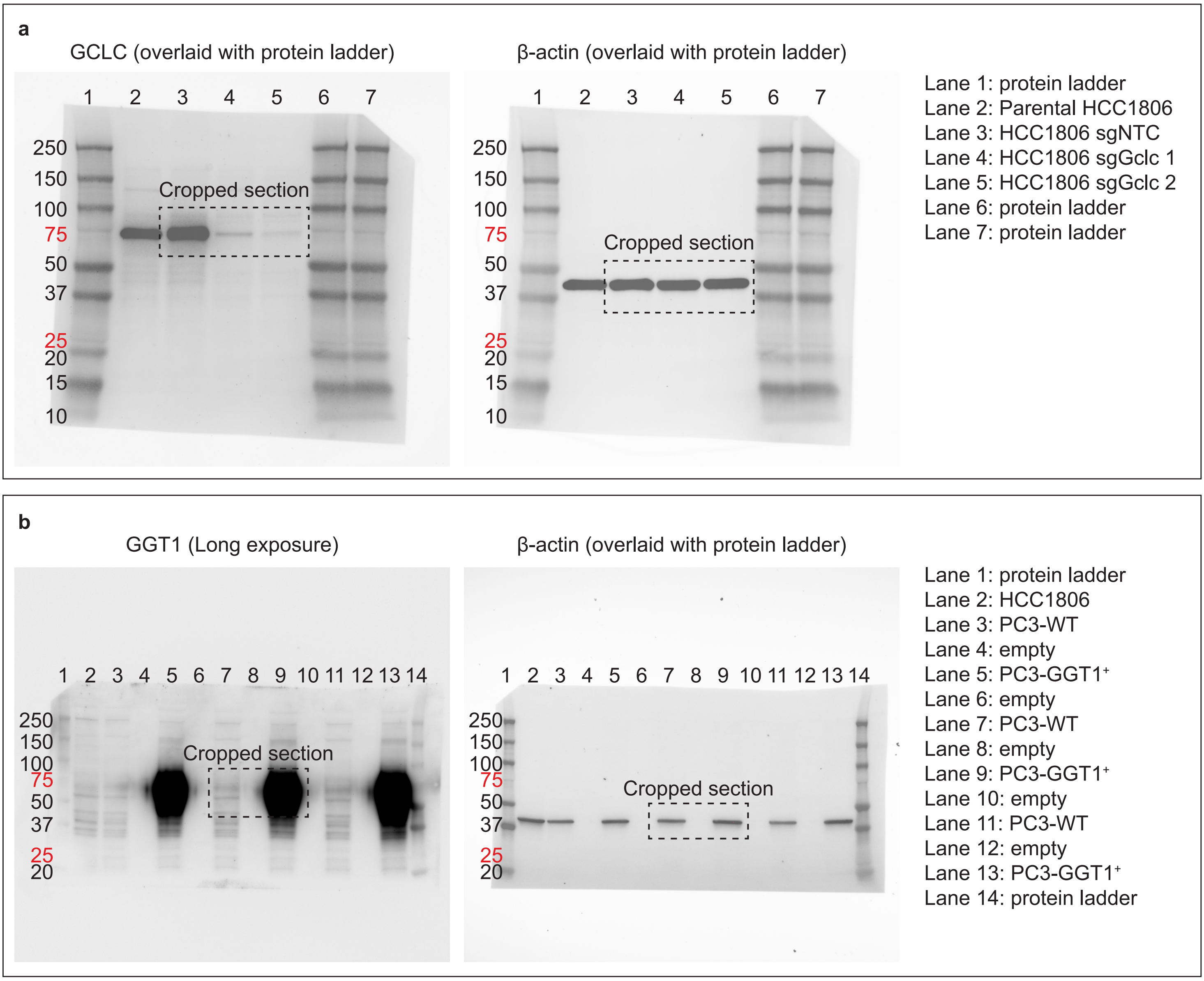

Supplementary Figure 1. Uncropped western blot membranes corresponding to Figure 1f **(a)** and Figure 3a **(b)**. For both experiments, the same membrane was sequentially probed for the target protein (i.e., GCLC **(a)** or GGT1 **(b)**) and the loading control β-actin, with membranes stripped between probings.
