# Supplementary material for: Catabolism of extracellular glutathione supplies cysteine to support tumours
Source: Nature. 2026 Mar 18;653(8115):933–41. doi: 10.1038/s41586-026-10268-2 (PMC13190318; doi:10.1038/s41586-026-10268-2)
Supplement: Supplementary file 2 — Reporting Summary [file 41586_2026_10268_MOESM2_ESM.pdf]

nature portfolio

Corresponding author(s): Fabio Hecht and Isaac Harris

Last updated by author(s): 01/26/2026

Reporting Summary

Nature Portfolio wishes to improve the reproducibility of the work that we publish. This form provides structure for consistency and transparency in reporting. For further information on Nature Portfolio policies, see our Editorial Policies and the Editorial Policy Checklist.

Statistics

For all statistical analyses, confirm that the following items are present in the figure legend, table legend, main text, or Methods section.

n/a	Confirmed	
☐	☒	The exact sample size (*n*) for each experimental group/condition, given as a discrete number and unit of measurement
☐	☒	A statement on whether measurements were taken from distinct samples or whether the same sample was measured repeatedly
☐	☒	The statistical test(s) used AND whether they are one- or two-sided<br>*Only common tests should be described solely by name; describe more complex techniques in the Methods section.*
☒	☐	A description of all covariates tested
☒	☐	A description of any assumptions or corrections, such as tests of normality and adjustment for multiple comparisons
☐	☒	A full description of the statistical parameters including central tendency (e.g. means) or other basic estimates (e.g. regression coefficient) AND variation (e.g. standard deviation) or associated estimates of uncertainty (e.g. confidence intervals)
☐	☒	For null hypothesis testing, the test statistic (e.g. *F*, *t*, *r*) with confidence intervals, effect sizes, degrees of freedom and *P* value noted<br>*Give P values as exact values whenever suitable.*
☒	☐	For Bayesian analysis, information on the choice of priors and Markov chain Monte Carlo settings
☒	☐	For hierarchical and complex designs, identification of the appropriate level for tests and full reporting of outcomes
☒	☐	Estimates of effect sizes (e.g. Cohen's *d*, Pearson's *r*), indicating how they were calculated

Our web collection on statistics for biologists contains articles on many of the points above.

Software and code

Policy information about availability of computer code

Data collection	• Microscopy images of cell lines in multi-well plates were acquired using either the CellInsight CX5 HCS instrument (Thermo) with HCS Navigator Software (Thermo, version 6.6.2, build 8533) or the SparkCyto instrument (Tecan) with SparkControl software (Tecan, version 4.0).
Data analysis	• Statistical analysis was performed using GraphPad Prism (version 10.6.1).<br>• Illustrations (Fig 1a, 2a,h,j, 3e, 4a,c) were created using Adobe Illustrator (version 30.1).<br>• MAPS data was processed using R (version 4.1.1).<br>• Flow cytometry analysis was performed using FCS Express 7 Research (DeNovo Software, version 7.24.0030).<br>• LC-MS metabolite peaks identification was conducted using Compound Discover (v3.3, Thermo Scientific), Skyline software (Version 25.1), and El-Maven software (Version 0.12.0).<br>• Proteomics data was processed with DIA-NN software (version 1.9.2, https://github.com/vdemichev/DIANN).<br>• Cell counts of images acquired by CellInsight CX5 HCS platform (Thermo) were determined using HCS Navigator Software (Thermo, version 6.6.2, build 8533).<br>• Cell counts of images acquired by SparkCyto (Tecan) were determined using SparkControl (Tecan, version 4.0).

For manuscripts utilizing custom algorithms or software that are central to the research but not yet described in published literature, software must be made available to editors and reviewers. We strongly encourage code deposition in a community repository (e.g. GitHub). See the Nature Portfolio guidelines for submitting code & software for further information.

Data

Policy information about availability of data

All manuscripts must include a data availability statement. This statement should provide the following information, where applicable:

- Accession codes, unique identifiers, or web links for publicly available datasets
- A description of any restrictions on data availability
- For clinical datasets or third party data, please ensure that the statement adheres to our policy

Source data for all figures are provided with this paper. Uncropped western blot images are provided in Supplementary Figure 1.

Publicly available datasets used in this study:
• GTEX: https://gtexportal.org/home/ (Extended Data Fig 1a, 9g)
• TCGA, accessed through cBioPortal platform: https://www.cbioportal.org/datasets (Extended Data Fig 9g,f,h)
• Cancer Cell Line Encyclopedia (CCLE): https://depmap.org/portal/ccle/ (Extended Data Fig 9b,c,d)
• DepMap: https://depmap.org/portal/ (Extended Data Fig 9e)
• ENCODE database, accessed through NCBI-Gene portal: https://www.ncbi.nlm.nih.gov/gene/ (BioProject: PRJNA66167) (Figure 10c)

Research involving human participants, their data, or biological material

Policy information about studies with human participants or human data. See also policy information about sex, gender (identity/presentation), and sexual orientation and race, ethnicity and racism.

Reporting on sex and gender	The biological sex of all 16 deidentified biospecimens was categorized as female.
Reporting on race, ethnicity, or other socially relevant groupings	The cohort included 81% white participants, 6% black, and 13% with race not reported.
Population characteristics	A total of 16 subjects were enrolled, all of whom were female with a median age of 64 years (range 25 to 88). Neoadjuvant treatment was administered in 4 subjects (25%). The cohort included 81% White, 6% Black, and 13% with race not reported. Most tumors were invasive ductal carcinoma (94%) with 6% classified as other histology. Estrogen receptor positivity was observed in 81% of cases and progesterone receptor positivity in 62%. HER2 status was distributed as 0 in 56%, 1+ in 32%, 2+ in 6%, and 3+ in 6%. Tumor staging showed 44% T1 and 56% T2 with no T3 or T4 disease. Nodal staging included 38% N0, 38% N1, 6% N2, and 18% NX. All subjects were M0 at diagnosis with no metastatic disease. Tumor grade was 1 in 12%, 2 in 50%, and 3 in 38%.
Recruitment	Human biospecimens and deidentified clinical data were provided by the Wilmot Cancer Institute Biobank Shared Resource (BSR) at the University of Rochester.
Ethics oversight	Human biospecimens and deidentified clinical data were provided by the Wilmot Cancer Institute Biobank Shared Resource (BSR) at the University of Rochester. All samples were collected under Institutional Review Board-approved protocols (STUDY61977 and STUDY7108), and all subjects provided written informed consent.

Note that full information on the approval of the study protocol must also be provided in the manuscript.

Field-specific reporting

Please select the one below that is the best fit for your research. If you are not sure, read the appropriate sections before making your selection.

☒ Life sciences ☐ Behavioural & social sciences ☐ Ecological, evolutionary & environmental sciences

For a reference copy of the document with all sections, see nature.com/documents/nr-reporting-summary-flat.pdf

Life sciences study design

All studies must disclose on these points even when the disclosure is negative.

Sample size	For cell culture experiments, sample sizes were not chosen based on statistical methods. All cell culture experiments were repeated at least 2 independent times, each with n≥3 technical replicates (i.e., independent wells) per experimental condition, unless stated otherwise. This number was selected based on pilot experiments and past experience showing that ≥2 independent experiments consistently captures biological variability and is sufficient to detect reproducible and statistically significant differences in cell growth and metabolite abundance between experimental conditions.<br><br>For animal studies, sample sizes were not chosen based on statistical methods. The number of animals assigned per condition was selected to account for the variability of the examined phenotypes based on pilot experiment and past experience with the animal models (≥6 animals per experimental condition).<br><br>For human studies, sample sizes were not chosen based on statistical methods. All available human samples were collected within the study

time frame, yielding a total of 16 deidentified plasma and breast cancer tissue specimens for analysis.

Data exclusions	For in vitro and in vivo studies, the existance of outliers was tested using the Robust Regression and Outlier Removal (ROUT) method (Q=1%) in GraphPad Prism 10.6.1 software. For animal xenograft studies, injections that resulted in a tumor of less than 50 mm3 within the first 14 days were excluded (prior to animal group allocation).
Replication	All in vitro data reported in this study (including metabolomics) were repeated at least twice (independent experiments), each including a minimum of two technical replicates (i.e., independent wells). All in vitro experiments (including metabolomics) were similar between replicate independent experiments. For proteomics, additional repeats were not conducted due to the magnitude of the observed effect and logistics required for the experiment. All animal experiments were conducted ≥2 independent times, each with with at least 6 animals per condition. All in vivo experiments were similar between independent replicate experiments.
Randomization	Mice of desired strains were age-matched and assigned randomly to their treatment groups. For xenograft studies, animals were allocated into groups ensuring the mean, median and standard error of tumor size was similar across all groups. For in vitro cancer cell culture experiments, cells at similar passage number (passage <25) were seeded at equal densities across all conditions and plate form factors to minimize inter- and intra-experimental variability. Human biospecimens were not assigned to different experimental groups, so randomization was not required.
Blinding	Investigators were not blinded to group allocation during experiments due to technical limitations. All treatments and measurements were performed under the same conditions for all animals. Further, all samples were analyzed together and subjected to the same data processing.

Reporting for specific materials, systems and methods

We require information from authors about some types of materials, experimental systems and methods used in many studies. Here, indicate whether each material, system or method listed is relevant to your study. If you are not sure if a list item applies to your research, read the appropriate section before selecting a response.

Materials & experimental systems

n/a	Involved in the study	
☐	☒	Antibodies
☐	☒	Eukaryotic cell lines
☒	☐	Palaeontology and archaeology
☐	☒	Animals and other organisms
☒	☐	Clinical data
☒	☐	Dual use research of concern
☒	☐	Plants

Methods

n/a	Involved in the study	
☒	☐	ChIP-seq
☒	☐	Flow cytometry
☒	☐	MRI-based neuroimaging

Antibodies

Antibodies used

Primary antibodies used in Western Blotting:
• rabbit-anti-GGT1 (affinity-purified, #GGT129). This antibody was produced by Dr. Marie Hanigan (University of Oklahoma) and it is not commercially available. Reference: PMID 8813074
• rabbit-anti-GCLC (Sigma-Aldrich HPA036359)
• mouse-anti-beta actin (Sigma-Aldrich A1978, Clone AC-15)

Secondary antibodies used in Western Blotting:
• donkey-anti-rabbit IgG-HRP (Amersham/ECL, NA934)
• sheep-anti-mouse IgG-HRP (Amersham/ECL NA931)

Primary anitbodies used for immunohistochemistry (IHC):
• anti-GCSc (SantaCruz sc390811)
• anti-CD45 (Santa Cruz sc1178)
• anti-F4/80 (Cell Signaling Technology 70076)
• anti-DNA/RNA Damage (Abcam 62623)
• anti-Nrf2 (Abcam 31163)

Secondary anitbodies used for immunohistochemistry (IHC):
• Biotinylated goat anti-mouse IgG (Vector Labs BA-9200)
• Biotinylated goat anti-rabbit IgG (Vector Labs BA-1000)

Primary antibodies used for immunofluorescence:
• anti-GCSc (SantaCruz sc390811)
• anti-CD45 (Proteintech 31243-1-AP)

Secondary antibodies used for immunofluorescence:
• Goat anti-Mouse-Alexa Fluor 594 (Invitrogen A11005)
• Goat-anti-rabbit-AlexaFluor 488 (Invitrogen A11034)

Antibody used for BrdU staining:
• anti-BrdU-FITC (Biolegend 364104, clone 3D4)

Validation

Primary antibodies used in Western Blotting:
• anti-GGT1: validation performed by Dr. Marie Hanigan (University of Oklahoma). Western Blotting detected bands of the expected size (Reference: PMID 8813074). Further, tissue IHC staining intensity (PMID 8813074) correlates with mRNA expression levels (ENCODE database).
• anti-GCLC: (Sigma Aldrich HPA036359): Validated by IHC, Western Blotting (via Orthogonal RNAseq and Capture MS), and Protein array by the Human Protein Atlas (HPA) project. Manufacture's recommended usage for WB: 0.04-0.4 µg/mL. Manufacture's recommended usage for IHC: 1:50-1:200. Reference: https://www.proteinatlas.org/ENSG00000001084-GCLC/summary/antibody
• anti-Beta-actin (Sigma Aldrich A1978, Clone AC-15): validated by vendor for western blotting with human samples. Manufacture's recommended usage for WB: 0.5-1 µg/mL using cell extract of human foreskin fibroblasts or chicken fibroblasts. Reference: https://www.sigmaaldrich.com/US/en/product/sigma/a1978

Primary anitbodies used for immunohistochemistry (IHC):
• anti-GCSc (SantaCruz sc390811): according to the manufacturer, sc390811 (H-5) is recommended for detection of g-GCSc of mouse, rat and human origin by immunohistochemistry, including paraffin-embedded sections. Starting dilution 1:50, dilution range 1:50-1:500. Reference: https://datasheets.scbt.com/sc-390811.pdf or https://www.scbt.com/p/gamma-gcsc-antibody-h-5
• anti-CD45 (Santa Cruz sc1178): according to the manufacturer, sc1178 is recommended for detection of CD45 of mouse, rat and human origin by immunohistochemistry (including paraffin-embedded sections). Starting dilution 1:50, dilution range 1:50-1:500. Refence: https://datasheets.scbt.com/sc-1178.pdf or https://www.scbt.com/p/cd45-antibody-35-z6
• anti-F4/80 (D2S9R) (Cell Signaling Technology 70076): according to the manufacturer, 70076 is recommended for detection of F4/80 of mouse origin by immunohistochemistry (including paraffin-embedded sections). Recommended dilution: 1:125 - 1:500. Reference: https://www.cellsignal.com/products/primary-antibodies/f4-80-d2s9r-rabbit-monoclonal-antibody/70076
• anti-DNA/RNA Damage (Clone 15A3) (Abcam 62623): according to the manufacturer, 62623 recognizes 8-hydroxy-2'-deoxyguanosine, 8-hydroxyguanine and 8-hydroxyguanosine by immunohistochemistry. Recommended usage dilution: 1 mg/mL. Reference: https://www.abcam.com/en-us/products/primary-antibodies/dna-rna-damage-antibody-15a3-ab62623
• anti-Nrf2 (Abcam 31163): according to the manufacturer, 31163 is recommended for detection of NRF2 of human, mouse (predicted), rat (predicted), chicken (predicted), and cow (predicted) by immunohistochemistry (including paraffin-embedded sections). Recommended dilution: 1:100. Reference: https://doc.abcam.com/datasheets/inactive/ab31163/en-us/nrf2-antibody-ab31163.pdf

Primary antibodies used for immunofluorescence:
• anti-GCSc (SantaCruz sc390811): according to the manufacturer, sc390811 is recommended for detection of g-GCSc of mouse, rat and human origin by immunofluorescence. Starting dilution 1:50, dilution range 1:50-1:500. Reference: https://datasheets.scbt.com/sc-390811.pdf or https://www.scbt.com/p/gamma-gcsc-antibody-h-5
• anti-CD45 (Proteintech 31243-1-AP): according to the manufacturer, 31243-1-AP is recommended for detection of CD45 of mouse, rat and human origin by immunofluorescence. Recommended dilution: 1:50-1:500. Reference: https://www.ptglab.com/products/CD45-Antibody-31243-1-AP.htm

Antibody used for BrdU staining:
• anti-BrdU-FITC (Biolegend 364104, clone 3D4): validated by vendor for Intracellular Staining for Flow Cytometry (ICFC). Manufacture's recommended usage for flow cytometric staining: 5µL/10^6 cells in 100 µL staining volume. Reference: https://www.biolegend.com/fr-lu/products/fitc-anti-brdu-antibody-10623

Eukaryotic cell lines

Policy information about cell lines and Sex and Gender in Research

Cell line source(s)

• HT-1080 (CCL-121, Lot: 70048591), NCI-H522 (CRL-5810, Lot: 70070052), and SKMEL28 (HTB-72, Lot: 70056504) were purchased from the American Type Culture Collection (ATCC).
• HCT-116 was purchased from the National Cancer Institute Division of Cancer Treatment and Diagnosis (NCI-DCTD) (Vial designation: 0507662, Lot: 0507660-0507663).
• HCC1806 (ATCC, CRL-2335), MDAMB231 (ATCC, HTB-26), MDAMB468 (ATCC, HTB-132), and JIMT-1 (DSMZ; ACC-589) were donated by Dr. Joan Brugge (Harvard University).
• PC3 (ATCC, CRL-1435) was donated by Dr. Marie Hanigan (University of Oklahoma).
• PaTu8988s (DSMZ, ACC-204) and SUIT2 (JCRB, 1094) were donated by Dr. Stephano Mello (University of Rochester).
• H1299 (ATCC, CRL-5803) and A549 were donated by Dr. Brian Altman (University of Rochester).
• 786-O (ATCC, CRL-1932) and A498 (ATCC, HTB-44) were donated by Dr. Phillip Rappold (University of Rochester).

Authentication

The authenticity of HCC1806, MDAMB231, MDAMB468, JIMT-1, and A549 was confirmed by STR profiling. The authenticity of the remaining cell lines was not tested.

Mycoplasma contamination

All cell lines tested negative for mycoplasma contamination using MycoAlert Mycoplasma Detection Kit (Lonza LT07-418).

Commonly misidentified lines
(See ICLAC register)

None of the 15 cell lines used in this study appear on ICLAC's Register of Misidentified Cell Lines (Version 13, released 26 April 2024).

Animals and other research organisms

Policy information about studies involving animals; ARRIVE guidelines recommended for reporting animal research, and Sex and Gender in Research

Laboratory animals	• 12-18 weeks female C57BL/6 Gclc f/f mice were crossed with the MMTV-PYMT (Jackson Lab, #022974) and Rosa26-CreERT2 (Jackson Labs, #008463) mouse strains.<br>• 8-12 weeks female athymic nude NU/J (Jackson Labs, #002019) were used for orthotopic tumor allografts and xenografts.<br>• Mice were housed in standard individually ventilated cages connected to a filtered air circulation system under pathogen-free conditions. Animals were maintained on a 12 h light/12 h dark cycle, with lights on during the daytime. Housing rooms were kept at controlled temperature (18-26°C) and relative humidity (30–70%). Food and water were provided ad libitum.
Wild animals	No wild animals were used in this study.
Reporting on sex	Since the major animal models of this study were models of breast cancer, all animals used in this study were females. Sex of mice were determined at 14-28 days using protocols approved by the University Committee on Animal Resources at the University of Rochester Medical Center.
Field-collected samples	Study did not involve samples collected from the field.
Ethics oversight	All animal studies were performed according to protocols approved by the University Committee on Animal Resources at the University of Rochester Medical Center.

Note that full information on the approval of the study protocol must also be provided in the manuscript.

Plants

Seed stocks	N/A
Novel plant genotypes	N/A
Authentication	N/A
